# Chemical cartography identifies potential mechanistic, diagnostic, and therapeutic opportunities in RAG deficiency

**DOI:** 10.3389/fimmu.2026.1960496

**Published:** 2026-10-23

**Authors:** Gwendolyn Cooper, Ottavia M. Delmonte, Ian S. LaCroix, Dimana Dimitrova, Marita Bosticardo, Heather Kenney, Gloria Magro, Francesca Pala, Zixiao An, Jenna R. E. Bergerson, Alexandra F. Freeman, Jennifer R. Heimall, Steven M. Holland, Danielle Fink, Douglas B. Kuhns, Nathan T. Brandes, Eric Bohrnsen, Jade Uriarte, Benjamin Schwarz, Luigi D. Notarangelo

**Affiliations:** 1 Research Technologies Branch, Rocky Mountain Laboratories, National Institute of Allergy and Infectious Diseases, National Institutes of Health, Hamilton, MT, United States; 2 Laboratory of Clinical Immunology and Microbiology, National Institute of Allergy and Infectious Diseases, National Institutes of Health, Bethesda, MD, United States; 3 Center for Immuno-Oncology, National Cancer Institute, National Institutes of Health, Bethesda, MD, United States; 4 Division of Allergy and Immunology, Children’s Hospital of Philadelphia, Philadelphia, PA, United States; 5 Neutrophil Monitoring Lab, Leidos Biomedical Research, Inc., Frederick National Laboratory for Cancer Research, Frederick, MD, United States

**Keywords:** hematopoietic stem cell transplantation, immunolipidomics, immunometabolomics, phenotypic heterogeneity, RAG deficiency

## Abstract

**Background:**

Mutations in the recombination-activating genes *(RAG1* and *RAG2*) in humans drive an array of immunodeficiencies with non-continuous symptom patterns. Previous multi-omic studies identified immune cell types and cytokines that defined RAG subtypes, but mechanisms remain to be established.

**Objective:**

To use circulating metabolic and lipidomic patterns to further resolve drivers of symptom heterogeneity and suggest alternative therapeutic targets in *RAG*-mutated patients.

**Method:**

*RAG*-mutated patient and healthy control plasma was analyzed across two cohorts using a chemical cartography approach wherein oxidative, dysbiotic, energetic, and neuroimmune patterns were broadly targeted through a series of overlaid immunometabolic and immunolipidomic profiling methods.

**Results:**

Patterns in tryptophan metabolism, purine metabolism, lipid stress molecules, and oxylipin products of 12-lipoxygenase segregated disease subtypes and presented potential targets in RAG-disease pathology. Purine metabolism and certain lipid oxidation phenotypes were exacerbated after hematopoietic stem cell transplantation, suggesting continuing pathology after treatment.

**Conclusions:**

This investigation reveals potentially targetable immune-related metabolic and lipidomic correlates of disease heterogeneity in human RAG-deficiency.

## Introduction

Heterogeneous disease presentation creates clinical challenges at the level of diagnosis and treatment. An illustrative case is the spectrum of disease caused by partial or complete loss of function in the recombination-activating genes (*RAG1* and *RAG2*) (1, 2), that encode for lymphoid-specific proteins that initiate the process of V(D)J recombination in the diversification of T and B cell receptors. Biallelic loss-of-function mutations in the *RAG* genes lead to complete arrest of B- and T-cell development resulting in severe combined immune deficiency disease (SCID) and a type 1 immune profile of blood and tissues (3, 4). Omenn syndrome (OS) is a severe near complete loss-of-function disease subtype defined by T helper 2 (T_H_2) skewed autoinflammatory symptoms including early-onset generalized erythroderma, lymphadenopathy, hepatosplenomegaly, eosinophilia and severe hypogammaglobulinaemia with increased IgE levels (3). Hypomorphic mutations in RAG1 and 2 lead to an array of immunopathologies collectively referred to as partial RAG Deficiencies (pRD) (5). Though previously divided into Leaky SCID (LS) and delayed onset combined immune deficiency (CID) subtypes, pRD is now considered as a single group due to symptom overlap and difficulties in classification (6). The heterogeneity in the clinical presentation and outcomes of human RAG deficiency, and pRD in particular, highlights the need for improved mechanistic understanding of the systemwide implications of RAG dysfunction.

Recently, our group has pursued a multiomic immune analyses of *RAG*-mutated patient cohorts including pRD, OS, and SCID assaying either the gut or circulation (3, 7). These studies identified signatures of immune dysregulation across RAG-disease subtypes that describe variance between individual patients; however, further analysis is needed to begin to identify mechanisms of these pathologies (3, 7). Immunometabolic and immunolipidomic profiling are emerging strategies for characterizing disease at the chemical level to identify symptom-associated processes and mechanisms that may be poorly resolved or invisible at the genetic or protein level (8–10).

Here, we examine the heterogeneity of RAG-disease presentation using a chemical cartography approach consisting of methodology calibrated to resolve key landmarks across the immunometabolic and immunolipidomic landscape. We identify patterns among tryptophan (Trp) metabolism and purine degradation via xanthine oxidoreductase (XOR), stress-associated lipids, and circulating oxidized immune lipid mediators (LMs) as both potential descriptors of disease subtypes and likely correlates of specific immune processes. Lastly, we show that allogeneic hematopoietic stem cell transplant (HSCT) reverses select chemical pathologies but exacerbates others including XOR and certain LMs, suggesting a residual immunometabolic dysregulation in these patients that may be targetable.

## Materials and methods

### Cohort design

Two groups of *RAG*-mutated patients, enrolled in protocols NCT03394053 and NCT03610802, approved by the NIH Institutional Review Board, and representing a main and a reproducibility cohort, were included in the study, for a total of 69 patients, 4 of which were included in both cohorts. Of the 69 patients, 37 were diagnosed with pRD, 12 with OS, 14 with SCID, and 6 patients were asymptomatic at the time of sample collection. The diagnosis of RAG deficiency was based on the demonstration of bi-allelic variants in either *RAG1* or *RAG2* gene, and the pathogenicity of the variants were demonstrated by an *in vitro* recombination assay (3). Diagnostic criteria for SCID, OS and pRD were as previously reported (11, 12). Eleven of the pRD patients included in the first cohort received HSCT, using either reduced-intensity, T-replete, post-transplantation cyclophosphamide-based (n=9) (13) or TCR alpha/beta-depleted (n=2) approaches. Plasma samples were collected at various time points after HSCT.

### Sample extraction

For all liquid chromatography – mass spectrometry (LC-MS) methods LC-MS grade solvents were used. All samples were processed via a modified Bligh-Dyer as previously described and diluted as necessary for injection (7). Additional details are provided in the **Supplementary Materials**.

### Metabolomics, lipidomics, and LM profiling

All metabolite, lipid, and LM measurements were profiled using in-house methods described in detail in the **Supplementary Materials**.

### Statistical methods

All signals were visually confirmed prior to integration. Data was filtered, normalized and scaled as described in the **Supplementary Materials**. Plots and analysis were generated using a combination of PRISM software (version 10.2.0) and R (version 4.4.1). The igraph package in R which was adjusted and compiled to generate and display correlation networks (https://github.com/IDSS-NIAID/MetLipNet). Matrix bar plots were assembled using the in-house developed matrixBP package.

## Results

### Cohort details and analytical strategy

To maximize clinical translation of these analyses, a chemical cartography approach was utilized, consisting of a series of broad, targeted, liquid chromatography tandem mass spectrometry (LC-MS/MS) methods calibrated to prioritize high-sensitivity and high-confidence measurement of key immunometabolic and immunolipidomic targets (7, 14, 15). Plasma samples were analyzed separately for polar metabolomics, lipidomics, and oxidized lipid mediators (LMs). Two separate but overlapping cohorts of patient plasma were analyzed over a year apart. Throughout our analysis, we chose to display the larger and more recent of these cohorts (Main) in the primary figures and use analysis of the smaller (Reproducibility) cohort to assess the robustness of patterns identified in the main cohort. Due to method progression, Main Cohort data included a broader breadth of metabolite and LM targets. In addition, lipidomics was applied only to the Main Cohort. Pediatric controls were included in both cohorts to account for the younger age of OS and SCID patients. Due to sample decay patterns evident in pediatric controls for both cohorts, which were collected at a much earlier date than the rest of the samples, these controls were excluded from statistical analysis, but all patterns identified here were subsequently assessed for robustness against those pediatric controls (further details in the **Supplementary Materials**).

Demographic data of patients and description of metabolite analysis are summarized in **Figure 1A-C** for the Main and Reproducibility Cohorts. pRD patients had much higher variations in age at study (6 weeks to 42 years old) when compared to patients with OS and SCID (2 months to 1 year old) (**Figures 1A, B**). Distinctive soluble biomarker profiles were observed in SCID, OS, and pRD patients (**Figures 1D, E**), confirming what was previously reported (3). A history and frequency of infections was present across all groups, with granulomas and other autoimmunity being predominantly descriptive of pRD patients whereas skin rash and severe viral infections were more common in OS and SCID (**Figures 1F, G**).

**Figure 1:**
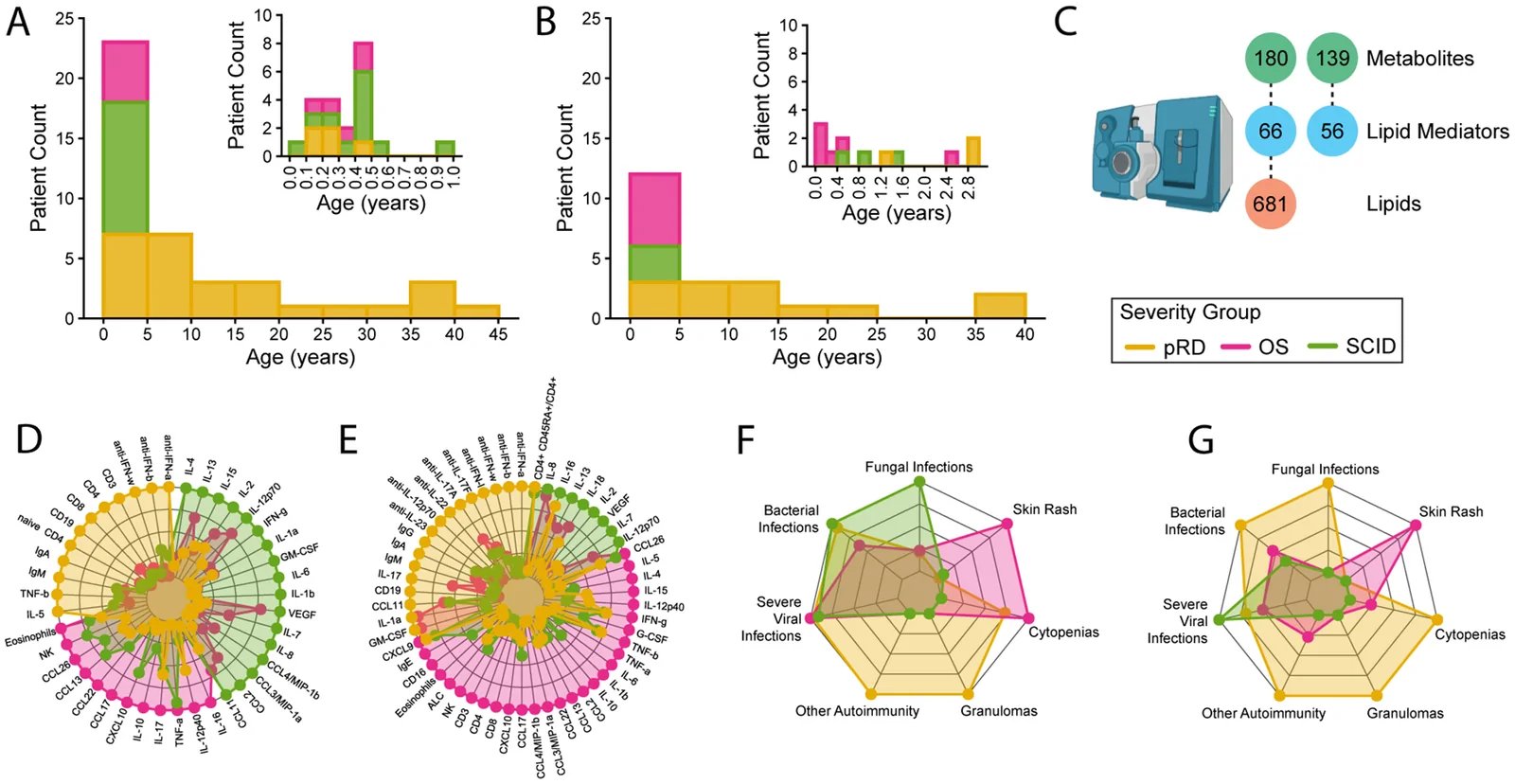
Immune processes, symptoms, and age separate RAG-disease subtypes. RAG severity groups were identified as pRD (yellow), OS (magenta), and SCID (green). Distribution of patient age within the **(A)** main cohort and **(B)** reproducibility cohort, broken down by RAG severity group. Inset panels highlight RAG severity for **(A)** ages 0–1 year and **(B)** 1–3 years. **(C)** Targeted mass spectrometry analyses identified 180 metabolites, 66 lipid mediators, and 681 lipids in plasma samples collected from the main cohort. 139 metabolites and 56 lipid mediators were identified in plasma samples collected from the reproducibility cohort. Figure created with Biorender.com. Lipidomics analysis was not performed for the reproducibility cohort. Cytokine profiles and immune cell assay readouts for the **(D)** main cohort and the **(E)** reproducibility cohort scaled to the maximum average value within the cohort. Symptom distributions across the **(F)** main cohort and the **(G)** reproducibility cohort as percentage of patients scaled to the maximum between the disease subtypes (3).

### Tryptophan and purine metabolism represent OS-dominant metabolic pathologies

We first resolved metabolomics patterns that distinguish disease subtypes. An OS and SCID-dominant pattern was evident in metabolites of Trp and XOR (**Figure 2A**; **Supplementary Figure 1**). Trp patterns included metabolites from all three major branches of Trp metabolism: the Kynurenine (Kyn) pathway, the Serotonin (5-HT) pathway, and the Indole pathway. OS and SCID exhibited lower levels of 5-HT and indole metabolites with trending decreases in pRD (**Figure 2B**). A lower-intensity, OS-distinguishing trending increase in specific Kyn metabolites was also present (**Figure 2B**).

**Figure 2:**
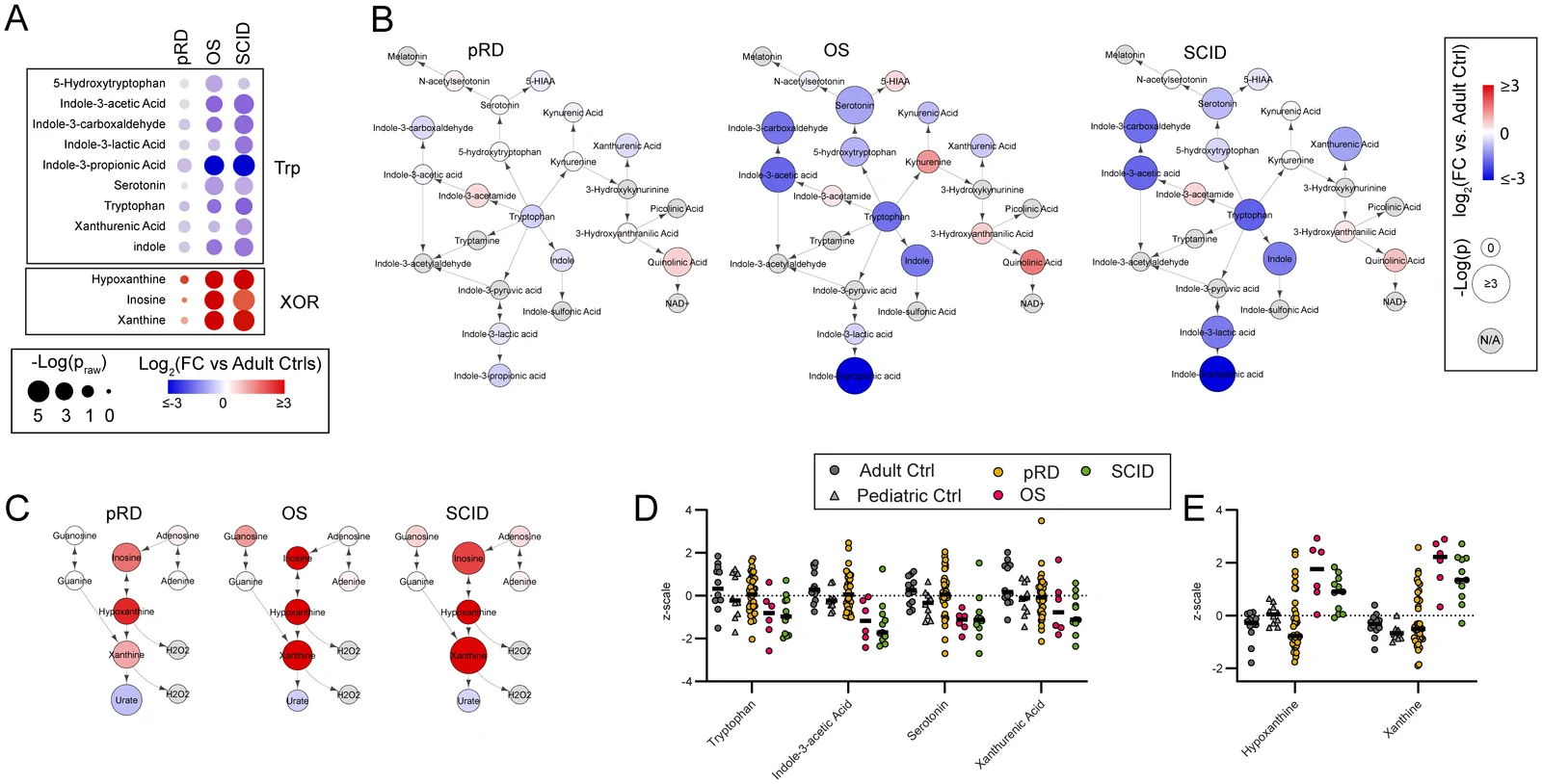
Metabolomics yields OS and SCID dominant patterns in Trp and purine metabolism. **(A)**. Results from Tryptophan (Trp) and Xanthine Oxioreductase metabolism from a comparison across RAG-disease subtypes and adult controls using a Kruskal-Wallis test. All metabolites displayed pass a correction for multiple comparisons at a 10% FDR via the Benjamini-Hochberg method and the full results are displayed in **Supplementary Figure 1**. **(B)** Trp metabolism map for each RAG-disease subtype compared to the adult healthy controls. **(C)** Xanthine oxidase metabolic maps for each RAG-disease subtype compared to the adult healthy control. **(D)** Scatter plots for representative metabolites from tryptophan metabolism including levels in pediatric control samples. The median of each group is marked. **(E)** Scatter plots of representative xanthine oxidase metabolites including pediatric control samples with the median of each group displayed. Data in **(D, E)** have been log-transformed and z-scaled for visualization. For **(A, B)**, and **(C)** the size of each node displays the negative logarithm of the raw p-value for each subtype compared to the adult healthy control samples via a Mann-Whitney U test. The color of each node displays the base two logarithm of the fold change of the indicated subtype relative to the adult healthy controls.

XOR patterns consisted of trending increases in both xanthine and hypoxanthine in pRD and became pronounced in SCID and OS with the highest levels reached in OS patients (**Figure 2C**). XOR catalyzes the conversion of hypoxanthine to xanthine and the subsequent conversion of xanthine to urate, both of which can produce reactive oxygen species (ROS) (16). Elevation of urate, which is a circulating marker of inflammatory activation, was absent (14). When compared to pediatric controls, both the Trp (**Figure 2D**) and XOR (**Figure 2E**) patterns were robust for OS and SCID.

Parallel univariate and mapped pathway analysis in the Reproducibility Cohort recapitulated the Trp and XOR phenotypes seen in the Main Cohort (**Supplementary Figures 2A–C**). Also similar to the Main Cohort, both the Trp and XOR patterns remained robust when compared to pediatric controls (**Supplementary Figure 2D**).

### Circulating lipidomics highlights RAG-associated stress phenotypes

A similar statistical strategy in the lipidomic data produced a series of lipid-class associated patterns that varied between disease subtypes including a pRD-dominated loss of neutral lipids, OS- and SCID-associated increases in lysophospholipids and sphingolipids, and a complex pattern between disease subtypes among phosphatidylcholine (PC) and phosphatidylglycerol (PG) (**Figure 3A**). To resolve variable within-class patterns, PC lipids were reorganized by the presumed the presumed sn-2 acyl, which was unambiguous for ether-linked PCs. For ester-linked PC series we defined the likely sn-2 acyl as the longer or more unsaturated acyl (**Figure 3B**) (17). RAG-disease patterns in PCs showed a clear loss of unsaturated acyls, including polyunsaturated fatty acids (PUFA), in both OS and SCID samples while pRD patient levels were unchanged or increased for these same lipids (**Figure 3B**).

**Figure 3:**
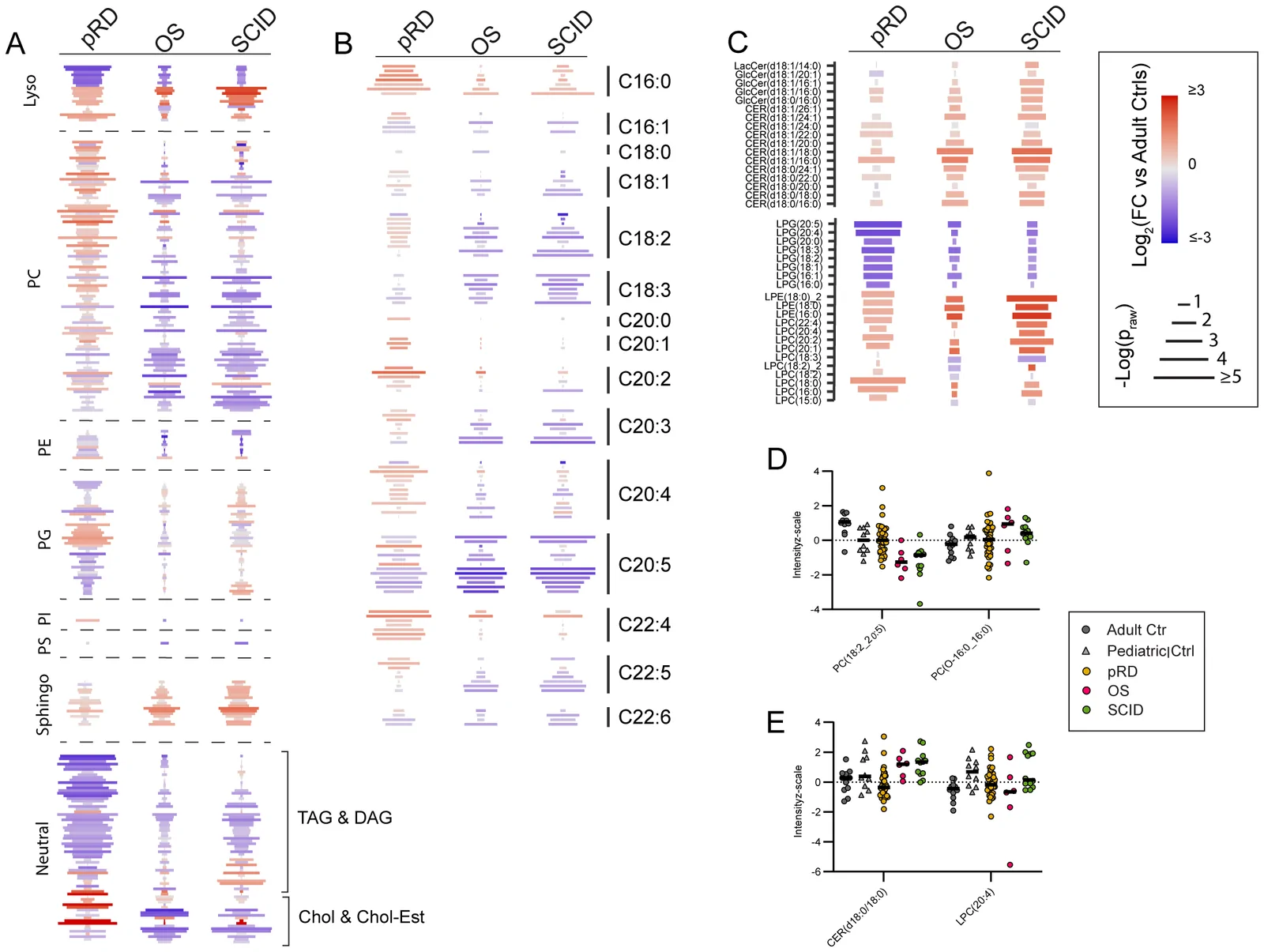
Circulating lipidomics reveals RAG-associated PUFA and lipid stress phenotypes. **(A)** Matrix bar plot of lipid comparison across RAG-disease subtypes and adult controls using a Kruskal-Wallis test. Lipids displayed pass a correction for multiple comparisons at a 10% FDR via the Benjamini-Hochberg method. Lipids are organized by lipid class. **(B)** Zoomed matrix bar plot of phosphatidylcholine (PC) including ester- and ether-linked PCs organized by the most likely acyl chain occupying the sn-2 position of the backbone glycerol as defined by longer and/or more unsaturated acyl. **(C)** Zoomed matrix bar plot of sphingolipids and lysophospholipids organized by lipid group. **(D)** Scatter plots of representative PCs including pediatric control samples with the median of each group displayed. **(E)** Scatter plots of representative lysophospholipids and ceramides including pediatric control samples with the median of each group displayed. Plots in **(A–C)** show the average base two logarithm of the fold change as line color and the negative logarithm of the Mann-Whitney U raw p-value as line length for each group vs. the adult controls. Data in **(D, E)** have been log-transformed and z-scaled for visualization. Lysophospholipids (Lysos), Phosphatidylcholine (PC), Phosphatidylethanolamine (PE), Phosphatidylglycerol (PG), Phosphatidylinositol (PI), Phosphatidylserine (PS), Sphingolipids (Sphingo), Triacylglycerol (TAG), Diacylglycerol (DAG), Cholesterol (Chol), Cholesterol-Ester (Chol-Est).

Known lipid stress patterns also differentiated RAG-disease subtypes via the patterns in sphingolipids and lysophospholipids (**Figure 3C**). Lysophospholipids and ceramides, which most strongly contributed to the sphingolipid patterns seen here in SCID and OS, are known circulating products, signals, and mediators of inflammation, stress, and ROS responses (18–22).

Unlike metabolomics results, which included muted patterns in pRD and either OS-SCID equivalent or OS-dominant immunometabolic phenotypes, lipid patterns did not directly align with the level of RAG activity remaining in the patient (**Figure 3A**) (3). Among certain neutral lipids, including triacylglycerols, diacylglycerols, pRD patients were dominant, while free and esterified cholesterol varied considerably between disease subtypes (**Figure 3A**). Among PCs, pRD samples showed elevation of arachidonic (C20:4) and docosatetraenoic (C22:4) acid-containing lipids, both of which were absent in OS and SCID (**Figure 3B**). Lysophospholipids and ceramides both reached their highest levels in SCID patients but certain lysophospholipids patterns were more defined in pRD than in OS (**Figure 3C**).

To confirm that these patterns were robust relative to pediatric controls, we plotted representative lipids from PCs, lysophospholipids, and ceramides across all disease and control groups (**Figures 3D, E**). Of the patterns described above, PUFA and ceramide patterns in each RAG-disease subtype were robust when compared to their respective control (adults for pRD and pediatric for OS and SCID) despite shifts in these lipids in the pediatric controls (**Figures 3D, E**). Lysophospholipid elevation was not resolvable in SCID or OS when compared to pediatric controls but the observed elevation in pRD patients compared to adult controls remained robust (**Figure 3E**).

### Oxidized immune lipid mediators suggest 12-LOX activity as a correlate of RAG-deficiency

The lipidome directly interacts with immune activation through the complex use of lipids as structural, energetic, and oxidative metabolites as well as direct signaling ligands. One such connection consists of the enzymatic or nonenzymatic oxidation of PUFAs into oxidized immune lipid mediators (LMs) (23). Consistent with disease-associated oxidative patterns in our metabolomics and PUFA-patterns in the lipidomics analysis, free-PUFAs and LM products of cyclooxygenase (COX), 12-lipoxygenase (12-LOX), and 15-lipoxygenase (15-LOX) readily distinguish disease subtypes (**Figure 4A**; **Supplementary Figure 3**). Free-PUFAs were decreased in pRD but not OS and SCID. Additionally, increases in LMs and LM-pathway markers produced by 5-lipoxygenase (5-LOX), 15-LOX, COX, and Cytochrome p450 (CYP450) were most pronounced in pRD patients (**Figure 4A, B**; **Supplementary Figure 3**). In contrast, 12-lipoxygenase (12-LOX) LMs and LM-pathway markers were most elevated in SCID and OS (**Figures 4A,B**). This suggested that pRD drives a generalized lipid oxidation pathology distinct from SCID and OS, which both are associated with a 12-LOX-specific chemical pathology.

**Figure 4:**
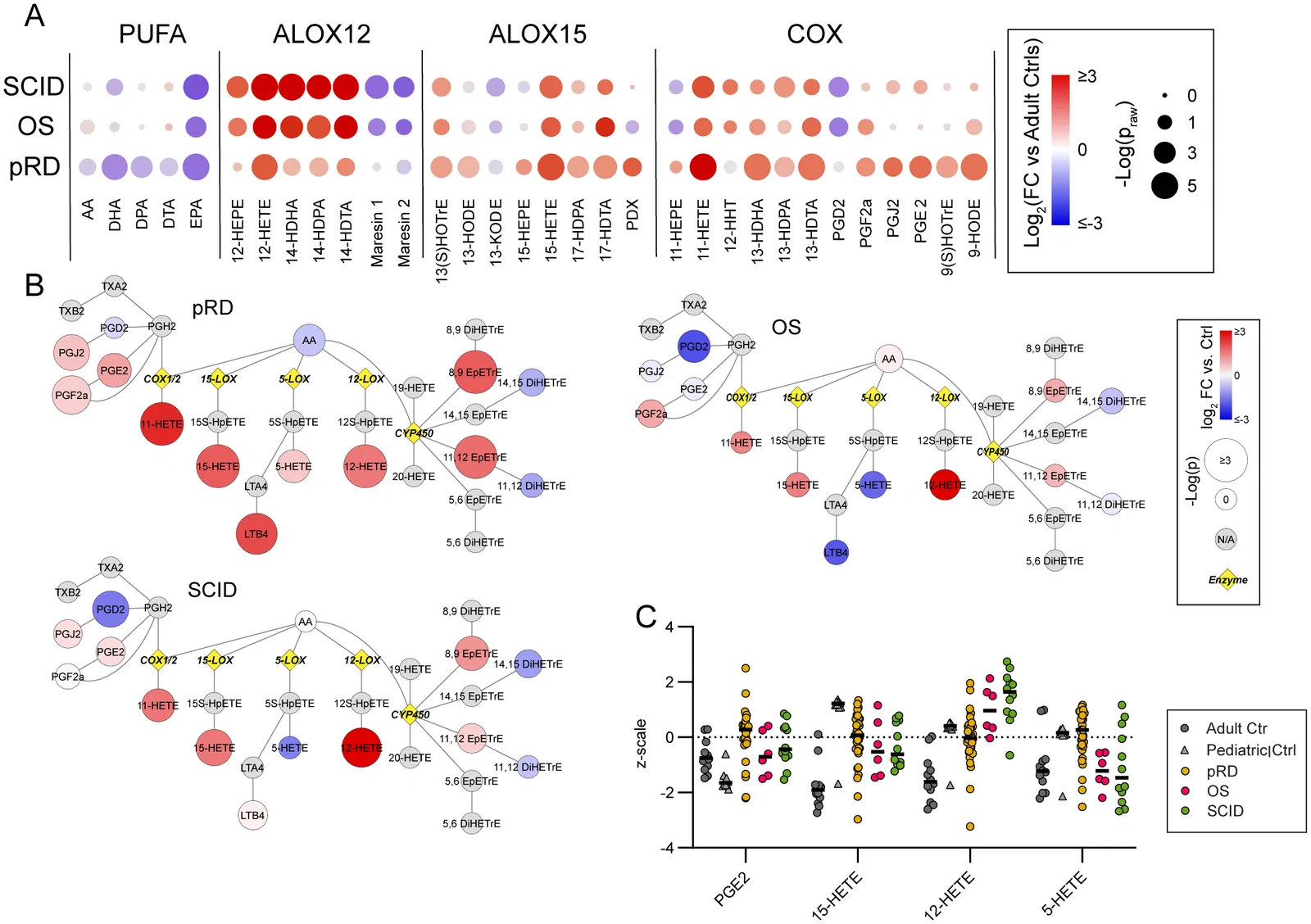
PUFA-derived LMs implicate ALOX12 activity as a correlate of RAG-deficiency. **(A)** Cross group LM comparison for select LM families across RAG-disease subtypes and adult controls from the main cohort using a Kruskal-Wallis test. LMs displayed pass a correction for multiple comparisons at a 10% FDR via the Benjamini-Hochberg method. LMs are organized by the enzymatic process that produces them. Complete results are displayed in **Supplementary Figure 3**. **(B)** Pathway maps of Arachidonic acid (AA) with LM metabolites shown as circles and enzymes shown as hexagons. **(C)** Scatter plots of representative LMs including in pediatric control samples with the median of each group displayed. Plots in **(A, B)** show the average base two logarithm of the fold change as node color and the negative logarithm of the Mann-Whitney U raw p-value as node size for each group vs. the adult controls. Data in **(C)** have been log-transformed and z-scaled for visualization. Polyunsaturated fatty acid (PUFA), Cyclooxygenase (COX), 12-lipoxygenase (12-LOX), and 15-lipoxygenase (15-LOX).

When compared to pediatric controls, 12-LOX patterns in SCID and OS were robust (**Figure 4C**). The Reproducibility Cohort recapitulated the all-form LM elevation in pRD patients, but this pattern was also shared by SCID and OS (**Supplementary Figure 4**). 12-LOX was elevated in SCID and OS patients in the Reproducibility Cohort but the pattern was not exclusive to that LM family.

### T-cell numbers, anti-interferon immunoglobulins, IL-7, and IL-8 correlate with separable chemical pathologies

We next assessed the utility of these omics data to describe immune variance across disease subtypes using a series of pairwise, multi-omic correlation networks (**Figure 5**). To avoid oversampling, lipidomic data was analyzed separate from metabolomics and LM data. Previous assessment of these same patients identified disease sub-type distinguishing patterns including higher autoreactive antibodies to interferons in pRD, elevated eosinophils, IgE, and cytokines in OS, and a specific subset of cytokines including IL-6, IL-7, and IL-8 in SCID (3). Assessing immune-correlated metabolite and LM molecules, a series of clusters consisting of LMs, metabolites, or a mixture of both was distinguishable (**Figure 5A**). The major visible metabolite separable cluster consisted of metabolites in addition to sarcosine, alanine, and 5-oxoproline (**Figure 5B**). Proximal to this metabolite cluster was an LM cluster consisting of COX and CYP450 products attached to several broadly networked XOR metabolites in the central immune cluster (**Figure 5B**). Both clusters were anchored to the immune measurements via direct or indirect correlation with IL-7 and IL-8 but also included CCL3 and IFN-γ. Separate from this primary cluster of metabolites, 12-LOX LMs and a series of metabolites including taurine, creatinine, serine, and sialic acid were anchored to the central IL-7/IL-8 hub, but also included correlations with IL-6, anti-interferon immunoglobulins (anti-IFN-ω and anti-IFN-α) and IL-1b (**Figure 5C**).

**Figure 5:**
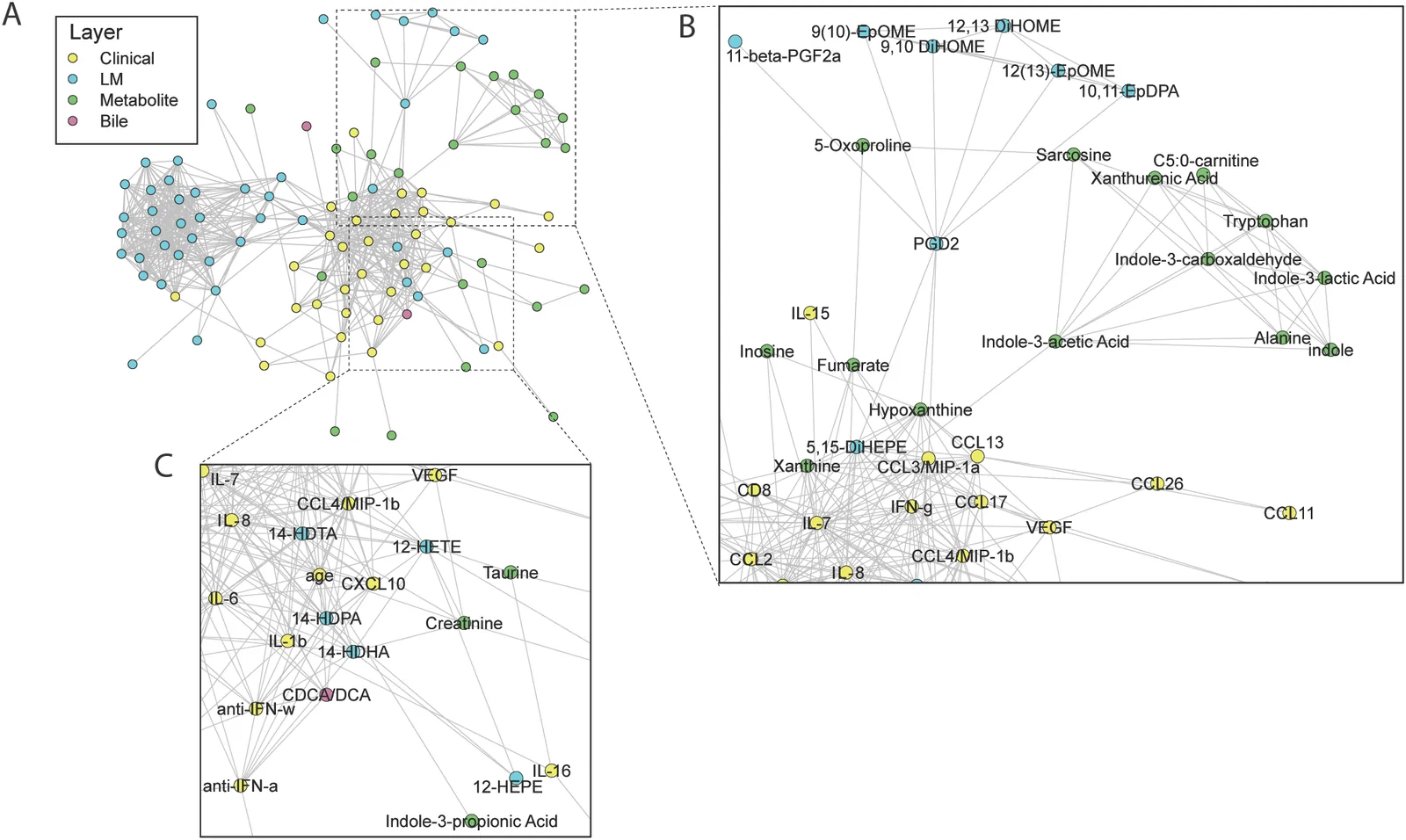
XOR-, Trp-, and 12-LOX-metabolites correlate with disease segregating immune measures: Pairwise nonparametric correlation network analysis of combined metabolomics and LM omics datasets from the main cohort mapping only first and second order neighbors of the RAG subtype critical element list (CD3, CD4, CD8, anti-IFN-α, anti-IFN-β, anti-IFN-ω, IL-6, IL-7, IL-8, IFN-γ, and CCL17). **(A)** Complete network map with all displayed connections reflecting an absolute value greater than 0.6 for the correlation coefficient and raw p-value less than 0.01. **(B)** Zoomed region of the same network shown in **(A)** showing Trp, XOR, and COX-associated patterns. **(C)** Zoomed region of the same network shown in **(A)** focused on LM metabolites of 12-LOX.

A similar network strategy, focused on lipidomic data, revealed two major lipid-class clusters including a PC-dominated cluster and a multi-class cluster around a central hub of immune measures (**Figure 6A**). The multi-class cluster included lysophospholipids, PGs, and TAGs and was anchored to the central immune cluster by associations to T-cell numbers (CD3, CD4, and CD8), IL-8 levels, and anti-IFN-α levels (**Figure 6B**). The TAGs contained in this cluster were well-represented for omega-3 PUFA docosahexaenoic acid (C22:6) (**Figure 6B**). The PC dominated cluster contained high numbers of both standard and ether-linked PCs with C20:4, C22:5, and C22:4 PUFAs overrepresented (**Figure 6C**). This last cluster was tightly associated with IFN-γ, IL-6, and anti-IFN-ω levels (**Figure 6C**).

**Figure 6:**
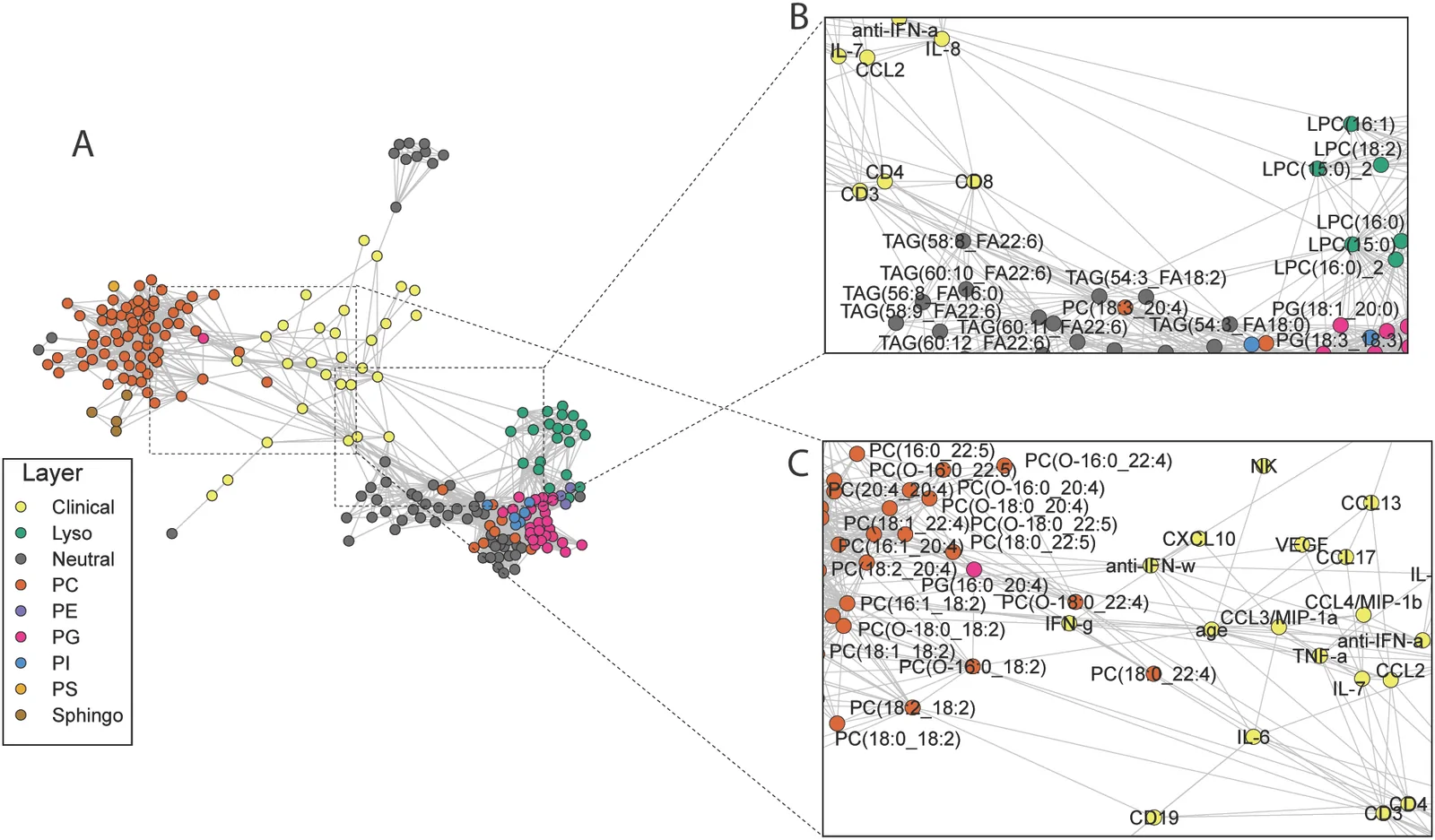
PUFA containing TAG and PC lipids correlate with immune measures: Pairwise nonparametric correlation network analysis of the lipidomic dataset from the main cohort mapping only first and second order neighbors of the RAG subtype critical element list (CD3, CD4, CD8, anti-IFN-α, anti-IFN-β, anti-IFN-ω, IL-6, IL-7, IL-8, IFN-γ, and CCL17). **(A)** Complete network map of with all displayed connections reflecting an absolute value greater than 0.6 for the correlation coefficient and p-value less than 0.01. **(B)** Zoomed region of the same network shown in **(A)** showing lipid patterns across lyso, TAG, and PG lipids associated with T-cell numbers, IL-8, and anti-IFN-α. **(C)** Zoomed region of the same network shown in **(A)** showing PC-localized PUFA interactions with IFN-γ, and anti-IFN-ω.

Network analysis was performed on the Reproducibility Cohort using a similar strategy (**Supplementary Figure 5**). Similar to the Main Cohort, a strong association of metabolic patterns, including Trp metabolites, with anti-IFN immunoglobulins was observed (**Supplementary Figure 5B**). An association with T-cell numbers was also observable here but was peripheral to the main clusters (**Supplementary Figure 5C**).

### HSCT in pRD patients reverses certain metabolic and lipidomic pathologies but exacerbates XOR activation and lipid oxidation

HSCT is the primary curative option for RAG deficiency. To determine if HSCT successfully reversed the metabolic and lipidomic pathologies observed here, pre- and post-HSCT plasma samples from pRD patients were compared across metabolomics, lipidomics, and LM datasets. For patients with repeated sampling after HSCT, each sample was considered separately due to powering limitations. Across all datasets, HSCT drove a reversal of markers of RAG-disease described above including a lowering of 5-oxoproline levels, increases in neutral lipids and PCs, decreases in stress-associated lysophospholipids and ceramides, and decreases in COX-produced LMs including the prostaglandins (**Figures 7A–C**). While not significantly affected, Trp-metabolites trended toward healthy control levels for all major Trp pathways (**Figure 7D**). Potentially exacerbated chemical pathologies were observable in both the metabolomics and LM datasets post-HSCT including further elevation of XOR metabolites and increases in mono-hydroxylated products of DHA (**Figures 7A,C, E, F**). The post-HSCT period proximal to transplant is known to involve dynamic recalibration of the immune system (24). Post-HSCT samples were collected between 2-weeks and 5-years post-treatment. To assess whether the XOR or LM exacerbation post-HSCT was localized to the early period of immune-recalibration, we binned post-HSCT samples into <1 month, 2–6 months, 7–12 months, 1–2 years, and 2+ years post-HSCT and compared these timepoints as the difference to the patient paired pre-HSCT value. No metabolite or LM features passed significance criteria for this analysis. Plotting the largest varying metabolite and LM that represent the residual XOR and LM signature post-HSCT (hypoxanthine and 7-HDHA) showed minimal trend in hypoxanthine and a continuing upward trend with time in 7-HDHA (**Figure 7G**). These results suggest that the dataset here cannot resolve time-dependent patterns if they exist under the powering limitations inherent.

**Figure 7:**
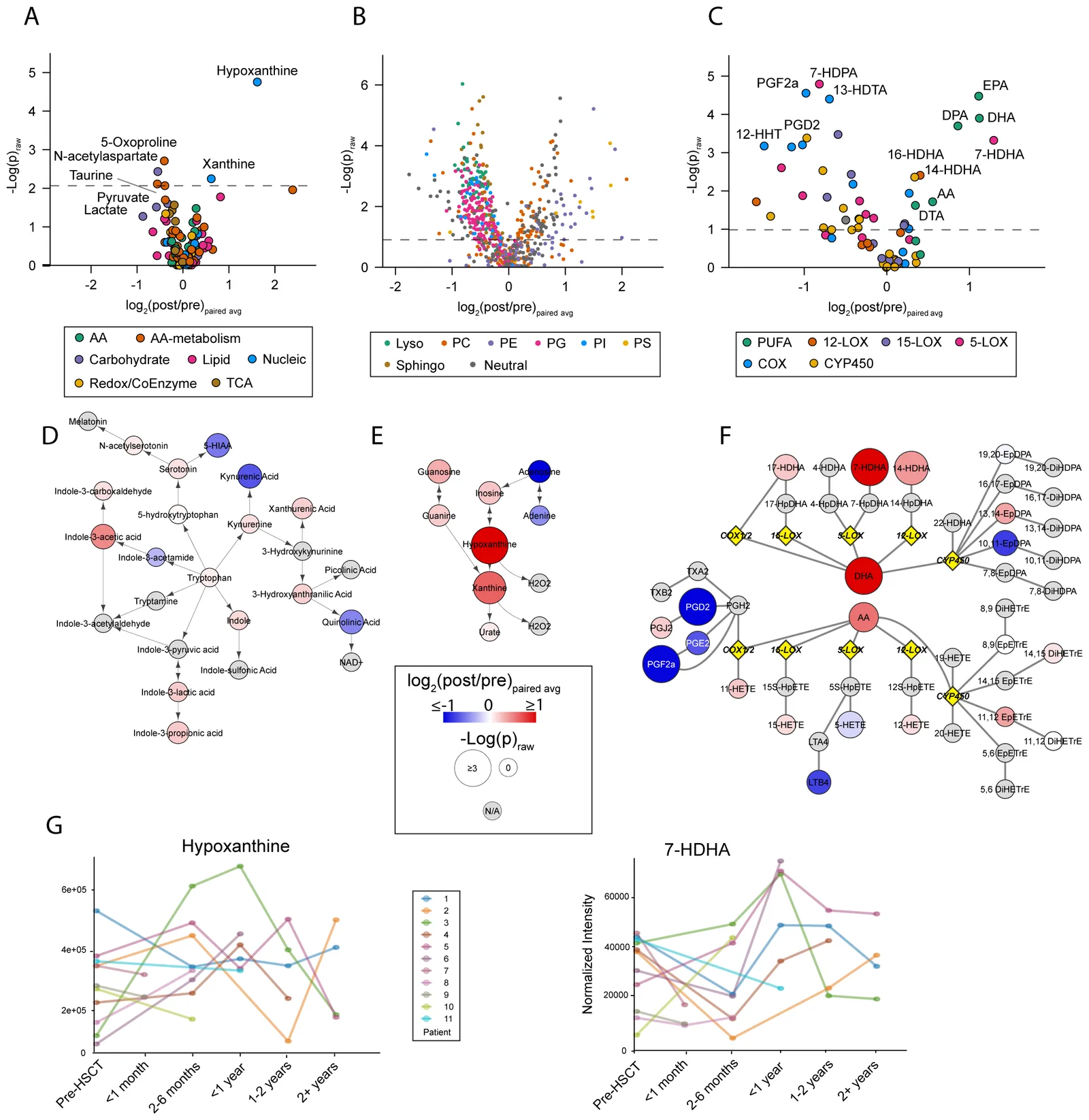
Allogeneic hematopoietic stem cell transplant (HSCT) partially reverses pRD-driven chemical pathologies. **(A-C)** Volcano plots displaying the results of non-parametric, paired post- vs. pre-HSCT plasma samples from pRD patients across the metabolomic, lipidomic, and LM datasets. **(D)** Pathway map of Trp metabolism showing changes among Trp-metabolites post-HSCT. **(E)** Pathway map of XOR metabolism showing changes among XOR-metabolites post-HSCT. **(F)** Pathway maps of both the AA and DHA LM and LM-pathway marker cascades post-HSCT. **(G)** Paired scatter plots of pRD patients across timepoints post-HSCT for a representative XOR and LM measure. Across **(D–F)** the size of each node displays the negative logarithm of the p-value for post-HSCT compared to pre-HSCT via a Mann-Whitney U test. The color of each node displays average, paired, base two logarithm of the fold change of post-HSCT relative to pre-HSCT samples in RAG-pRD patients.

## Discussion

Here, we expand on our previous multiomic investigation into the heterogeneity of RAG-disease presentation by mapping circulating immune-relevant metabolites, lipids, and immune lipid mediators (LM) (3). Within these data we identify Trp metabolism, XOR, stress-associated lipids, and LMs produced by 12-LOX as indicators of complete or near complete loss of RAG activity in OS and SCID patients. We find that pRD is marked by a combined lipid and LM chemical pathology that includes PUFA-containing phospholipids, neutral lipids, and higher levels of lipid oxidation across all LM pathways. Lastly, we identify residual chemical pathologies post HSCT-treatment in pRD individuals including exacerbation of metabolite and LM markers of immune driven ROS. These patterns serve as hypotheses for the further investigation of diagnostics and mechanistic insights into RAG disease heterogeneity.

Trp metabolism is a known hub where the immune system, nervous system, and the microbiome interact and regulate each other through multifunctional metabolites from each of the three major arms of Trp metabolism: the Kyn pathway, the 5-HT pathway, and the Indole pathway (25–30). The Kyn pathway is triggered by immune signaling, including by interferon-γ (IFN-γ), during inflammation and mediates immunosuppression and immunoregulation depending on the specific downstream metabolites produced (27, 28, 30). Markers of this pathway trended upward primarily in OS despite the Th2 bias of the immune response in those patients and the higher IFN-γ levels in SCID patients. Previous studies have also shown that prostaglandins can drive IDO1 expression, but these two processes did not interact in either cohort and were anticorrelated via network analysis between pRD (high PGE2, PGF2a, and PGJ2) and SCID/OS (higher Kyn-metabolites) (31). This misalignment of Kyn pathway marker levels and the conventional stimulus suggests an alternative route of pathway activation.

Changes in the Indole and 5-HT arms of Trp metabolism were more pronounced than changes in the Kyn pathway and both followed the level of residual RAG activity in the patients. The circulating pools of metabolites from both pathways heavily involve or exclusively rely on the microbiome for production (25, 26). Depletion of these metabolites in circulation, in addition to observed decreases in secondary bile acids, further supports previous work by our group highlighting gut dysbiosis as a key unifying pathology in RAG patients (7).

The final two steps of purine degradation from hypoxanthine to urate are facilitated by XOR. The specific metabolites consumed and produced by XOR are context and cofactor dependent, but under inflammatory conditions, it tends to generate ROS and reactive nitrogen species (RNS) as part of the inflammatory cascade (35, 36). Here, XOR metabolites are among the closest correlates of cytokine and cellular markers of RAG-disease subtypes suggesting that this pathway is a participant in disease. The highest levels of XOR metabolites observed in OS are consistent with our previous observation that the transcriptional profile of circulating CD4^+^ cells from OS patients is indicative of prominent ROS production (3).

While metabolomics patterns largely described the level of RAG activity in the patients, lipidomics and LM analysis revealed nonlinear patterns in chemical pathologies that were associated with specific immune processes and symptoms. In these data, pRD drove a separate series of lipidomic changes from those evident in SCID and OS. While patterns in PUFA-containing phospholipids suggested changes in lipid oxidation processes, including the generation of LMs, the implications of pRD-dominant neutral lipid patterns were less clear. In our multi-omic network analysis, a family of DHA-containing neutral TAGs clustered with T-cell numbers, which was driven by pRD patients as controls were not considered in that analysis due to the absence of cellular data (CD3, CD4, & CD8). This suggests that the neutral lipid pool in pRD patients could be a participant in the broader PUFA and lipid oxidation phenotype in these patients.

Patterns in PUFA oxidation and downstream LM generation were also complex correlates of RAG-disease subtypes. SCID and OS shared a 12-LOX specific pathology consisting of elevated 12-LOX monohydroxylated LM species and decreases in downstream immunoregulatory Maresin-1 and -2 LMs. This pattern closely correlated with multiple cytokine and cellular markers of disease as well as XOR metabolites. Like most of the LM-producing lipoxygenases, 12-LOX has a context-dependent role in immune-activation, regulation, and resolution (37). Given the inverse association between 12-LOX LMs and residual RAG activity, we suspect that 12-LOX may be a contributor to disease here. In cases of Th2 skewed immunity, such as those found in the OS patients, 12-LOX has been found to be a key regulator of the immune response via suppressive activity on group 2 innate lymphoid cells (ILC2s) (3). However, the elevated 12-LOX patterns seen in OS are misaligned with a ILC2-regulatory mechanism and, additionally, ILC2 expansion was exclusively observed in OS patients in this cohort despite the 12-LOX pattern being present in SCID patients with comparable magnitude (3). Due to the predominance of 12-LOX in platelets in the circulating environment, this 12-LOX pattern is anticipated to reflect platelet dysregulation in OS and SCID (38). Immune-mediated thrombocytopenia has been observed in human RAG deficiency, although more often in pRD than in OS or SCID (39). Further work will focus on assessing the strength and localization of this 12-LOX pathology in patients.

While defining in the Main Cohort and present in our Reproducibility Cohort, the specificity of the 12-LOX pattern in SCID and OS was not reproducible in the Reproducibility Cohort. The inability to reproduce this pattern reflects a limitation of studying oxidation products with differences in sample storage conditions and or time. The strength of the pattern-specificity in the Main Cohort suggests that further investigation is warranted to better map patient to patient variance in 12-LOX LMs and establish cellular localization of changes in 12-LOX expression. The involvement of 12-LOX in RAG pathology will require the identification of the specific cell type producing these compounds in circulation, and functional assays targeting that pathway in cellular or murine models.

This manuscript has some limitations. RAG-deficient patients are highly susceptible to acute and chronic infections, as well as to autoimmunity, which may require use of immunosuppressants. Because patients with such features were not excluded from the study, the possible confounding effect of these variables must be taken into account. Caution should be used in the interpretation of HSCT-associated metabolic changes, as a limited number of samples were collected over a broad interval since the time of transplantation, and the confounding effect of conditioning regimen used, quality of immune reconstitution, presence of active or chronic infections and use of immunosuppressive drugs could not be evaluated. Age of the patient is another strong confounder of both RAG-disease and metabolism. Relevant here given the RAG-disease associated Trp patterns identified, Trp metabolite levels have been shown to alter with age and sex in a manner that is dependent on the matrix analyzed (32). In humans, newborns can have 2–4 times higher circulating Trp than adults for the first three weeks post-partum but these levels are reduced to those of adults by the fourth week of life (33). Trp and Kyn levels have been shown to increase with age in mouse heart and liver while indole-3-acetic acid has been shown to decrease (34). Differences in sample storage time limited the statistical inclusion of our pediatric control samples toward assessing the degree to which the patterns described here were driven by disease or metabolic covariates of age (summarized in **Supplementary Material**). All patterns discussed here, with the exception of lysophospholipids elevation and the LMs in the Reproducibility Cohort, were still evident when viewed against the recovered pediatric control data but this does not definitively exclude age as a confounding variable. Further investigation is needed to better understand the relative contribution of patient age to potential RAG-disease associated chemical patterns described here.

This investigation was also limited by imperfect agreement between the two cohorts examined. While metabolomics patterns were well aligned across cohorts for both Trp and XOR metabolites, LM patterns observed in the Main Cohort, including the 12-LOX pattern, were not resolvable in the Reproducibility Cohort. This included the exclusive elevation of 12-LOX products in OS and SCID patients as well as the resolution of this pattern over the autooxidation effect apparent in the pediatric control samples (**Supplementary Figure 4C**). The cause of this difference is unclear but may arise from the lower powering of the Reproducibility Cohort or differences in the specific included patients. Regardless of the cause of this disagreement, the LM patterns identified here will require further investigation to facilitate confirmation.

pRD exhibited a non-specific increase in all LMs suggesting a different origin for this lipid oxidation in these patients compared to OS and SCID. Portions of this pRD LM pattern including monohydroxylated products of DHA were exacerbated post-HSCT despite reversal of COX-synthesized LMs such as the prostaglandins. With the early evidence of exacerbated XOR pathology post-HSCT, these residual LM oxidation patterns support a limited increase in oxidative stress in post-HSCT patients even years later, potentially explainable by the effects of chemotherapy and, in some cases, alloimmune complications (40). If this oxidative stress can be localized and addressed, it has the potential to further improve long-term outcomes for these post-treatment patients (41).

## Data Availability

The original contributions presented in the study are included in the article/**Supplementary Material**, further inquiries can be directed to the corresponding author/s.

## Glossary

5-LOX: 5-Lipoxygenase
12-LOX: 12-Lipoxygenase
15-LOX: 15-Lipoxygenase
AA: Arachidonic Acid
CID: delayed onset combined immune deficiency
COX: Cyclooxygenase
CYP450: Cytochrome P450
DAG: Diacylglycerol
DiHETrE: Dihydroxyeicosatrienoic Acid
DHA: Docosahexaenoic Acid
DPA: Docosapentaenoic Acid
DTA: Docosatetraenoic Acid
EET: Epoxyeicosatrienoic Acid
EPA: Eicosapentaenoic Acid
EpDPA: Epoxydocosapentaenoic Acid
EpOME: Epoxyoctadecenoic Acid
HDHA: Hydroxydocosahexaenoic Acid
HDPA: Hydroxydocosapentaenoic Acid
HDTA: Hydroxydocosatetraenoic Acid
HETE: Hydroxyeicosatetraenoic Acid
HEPE: Hydroxyeicosapentaenoic Acid
HODE: Hydroxyoctadecadienoic Acid
HSCT: Hematopoietic stem cell transplant
IFN-γ: interferon-γ
ILC2s: Type 2 innate lymphoid cells
Kyn: Kynurenine
LC-MS: Liquid chromatography
LS: Leaky SCID
LMs: Lipid mediators
LTB4: Leukotriene B4
Lysos: Lysophospholipids
PC: Phosphatidylcholine
PDX: Protectin DX
PE: Phosphatidylethanolamine
PG: Phosphatidylglycerol
PGD2: Prostaglandin D2
PGE2: Prostaglandin E2
PGF2a: Prostaglandin F2a
PGJ2: Prostaglandin J2
PI: Phosphatidylinositol
pRD: partial RAG deficiency
PS: Phosphatidylserine
PUFA: Free Polyunsaturated Fatty Acid
RNS: Reactive nitrogen species
ROS: Reactive oxygen species
SCID: Severe combined immune deficiency disease
Sphingos: Sphingolipids
TAG: Triacylglycerol
TCA: Tricarboxylic Acid Cycle
Trp: Tryptophan
OS: Omenn Syndrome
XOR: Xanthine Oxidoreductase.
