# Supplementary material for: Chemical cartography identifies potential mechanistic, diagnostic, and therapeutic opportunities in RAG deficiency
Source: Front Immunol. 2026 Oct 23;17:1960496. doi: 10.3389/fimmu.2026.1960496 (PMC13739484; doi:10.3389/fimmu.2026.1960496)
Supplement: Supplementary file 1 [file SupplementaryFile1.docx]

**Online Repository**

**Additional Method Details:**

*Cohort Design*

Eleven patients received allogeneic hematopoietic stem cell transplantation (HSCT). Of these, 9 were transplanted at the National Institutes of Health according to protocol NCT02579967 (n=8) or NCT03663933 (n=1) (clinicaltrials.gov). Age at HSCT ranged from 4 to 38 years, median 10 years. All patients received reduced intensity conditioning with a pentostatin/hyperfractionated cyclophosphamide/busulfan backbone, T-cell replete grafts, and post-transplantation cyclophosphamide (PTCy). Patients transplanted according to NCT02579967 received marrow with either high-dose PTCy (100mg/kg total, n=6) or reduced-dose post-transplantation cyclophosphamide (75mg/kg total, n=2), along with sirolimus +/- mycophenolate mofetil (MMF). The patient transplanted according to NCT03663933 received distal equine antithymocyte globulin in addition to the above conditioning backbone, with a peripheral blood graft, high-dose PTCy, MMF and tacrolimus. All but 2 donors were heterozygous RAG1 mutation carriers. All samples were stored at -80°C and did not undergo more than one freeze-thaw cycle until subsequent metabolomic and lipidomic analyses.

*Metabolite Sample Extraction*

For all LCMS methods, LCMS grade solvents were used. All samples were processed via a modified Bligh-Dyer extraction. All samples were immersed in a sample-matching volume of ice-cold methanol. To each sample a volume of chloroform was added. Samples were shaken for 30 minutes under refrigeration and centrifuged at 16k xg for 20 min. 400 µL each of the top (aqueous) layer and bottom (organic) were collected separately. The remaining aqueous layer was diluted 5x in 50 % methanol in water for LCMS analysis of central polar metabolites and oxidized lipid mediators. The organic layer was dried down under vacuum and resuspended in an equivalent volume of 5 µg/mL butylated hydroxytoluene in 6:1 isopropanol:methanol for targeted bulk lipidomics and bile acid analysis.

*Metabolomics*

*Main Cohort*

Metabolites were analyzed using an LD40 XR UHPLC (Shimadzu Co.) system for separation and a 6500+ QTrap mass spectrometer (AB Sciex Pte. Ltd.) for detection. Samples were separated on a Phenomenex Kinetex F5 column (100Å, 2.6 µm, 100 x 2.1 mm) and eluted using a binary gradient from 5mM ammonium acetate in 95% water pH 7.5, 5% acetonitrile to 8mM ammonium acetate in 95% acetonitrile, 5% pH 7.5 water over 5 minutes.

*Reproducibility Cohort*

Aqueous metabolites were analyzed using a combination of two analytical methods with opposing ionization polarities. Both methodologies utilized an LD40 XR UHPLC (Shimadzu Co.) system for separation and a 6500+ QTrap mass spectrometer (AB Sciex Pte. Ltd.) for detection. Negative mode samples were separated on a Waters™ Atlantis T3 column (100Å, 3 µm, 3 mm X 100 mm) and eluted using a binary gradient from 5 mM tributylamine, 5 mM acetic acid in 2% isopropanol, 5% methanol, 93% water (v/v) to 100% isopropanol over 5 minutes. Two distinct MRM pairs in negative mode were used for each metabolite. Positive mode method samples were separated across a Phenomenex Kinetex F5 column (100 Å, 2.6 µm, 100 x 2.1 mm) and eluted with a gradient from 0.1 % formic acid in water to 0.1 % formic acid in acetonitrile over 5 minutes.

*LMs, Main and Reproducibility Cohort*

Lipid mediators were analyzed using an LD40 XR UHPLC (Shimadzu Co.) system for separation and a 7500+ QTrap mass spectrometer (AB Sciex Pte. Ltd.) for detection. Samples were separated using an Phenomenex Kinetex Polar C18 column (100 Å, 2.6 µm, 3 mm x 100 mm) and eluted using a binary gradient from 0.01% acetic acid in water to 0.01% acetic acid in methanol over 8.7 minutes.

*Bile Acids Reproducibility Cohort*

Bile acids were analyzed using an LD40 XR UHPLC (Shimadzu Co.) system for separation and a 6500+ QTrap mass spectrometer (AB Sciex Pte. Ltd.) for detection. Samples were separated using an Phenomenex Kinetex Polar C18 column (100 Å, 2.6 µm, 3 mm x 100 mm) and eluted using a binary gradient from 0.01% acetic acid in water to 0.01% acetic acid in methanol over 4.5 minutes.

*Lipidomics*

Bulk lipidomics was performed on a LD40 X3 UHPLC (Shimadzu Co.) and a 7500 QTrap mass spectrometer (AB Sciex Pte. Ltd.) for separation and detection. Lipids were separated by lipid class on a Water XBridge Amide column (3.5 μm, 3 mm X 100 mm) using a 9-minute binary gradient from 100% 5 mM ammonium acetate, 5% water in acetonitrile apparent pH 8.4 to 95% 5 mM ammonium acetate, 50% water in acetonitrile apparent pH 8.0. All lipids were detected using scheduled MRMs that leveraged fatty acid product ions, headgroup product ions, or neutral loss ion combinations that were conserved within each lipid class.

*Statistical methods*

Blanks and quality control samples were injected every 10 samples throughout each run to track spectral drift and assess LCMS performance. All signals were integrated using SciexOS 3.1 (AB Sciex Pte. Ltd.). Signals with greater than 50% missing values for a specific dataset were discarded and remaining missing values were replaced with 1/5th the lowest registered signal value. Where appropriate, signals with a QC coefficient of variance greater than 30 % were discarded. Metabolites, lipids, and LMs with multiple MRMs were quantified with the higher signal to noise MRM. Metabolomics and lipidomics data were total sum normalized after initial filtering and LM datasets were normalized to the total sum of the metabolomics signal for the same sample to facilitate a more diverse foundation for normalization. All data were inspected manually to determine if any issues arose during the run that would require quality control correction, though none were found.

Plots and analysis were generated using a combination of PRISM software (version 10.2.0) and R (version 4.4.1). For multiple group comparisons, a Kruskal-Wallis test was applied followed by a Benjamini-Hochberg correction for multiple comparisons at the indicated false discovery rate (FDR) cutoffs. For two-group comparisons and displays, a Mann-Whitney U test was used followed by a Benjamini-Hochberg correction where indicated. To investigate relationships shared between small molecules measured in the multi-omic analyses and clinical covariates, a correlation-based network analysis was conducted. All clinical co-variates described in Figure 1, including age, cell counts, infection status, and cytokine profiles were included in the correlation analysis. These relationships were then visualized via the igraph package in R which was further adjusted and compiled for these datasets (https://github.com/IDSS-NIAID/MetLipNet). Matrix bar plots were assembled using the publicly available, in-house developed matrixBP package.

*Data coercion to account for metabolite decay due to differential time of sample storage:*

Pediatric controls were analyzed in both cohorts for comparison to SCID and OS patients, which were universally children or infants. Unfortunately, due to the difficulty of obtaining healthy pediatric samples, pediatric controls in both the main and reproducibility cohort were collected at earlier dates than either adult controls or patient samples. Metabolites, lipids, and LMs are known to oxidize or otherwise decay even under optimal storage conditions.(1, 2) To characterize the presence and magnitude of decay associated patterns, we analyzed each dataset using Principal Component Analysis (PCA) (Sup Fig 6, Sup Fig 7). Pediatric control-driven separation was evident in all three datasets in the main cohort (Sup Fig 6A-C, Sup Fig 7A-C). These signatures were localized to the loss of high energy molecules in the metabolomics data and oxidation signatures in both the lipidomics and LM datasets (Sup Fig 6D-F, Sup Fig 7D-F). To improve the confidence in the downstream metabolomic analysis, the metabolomics dataset was filtered to remove all phosphorylated or coenzyme containing molecules and the data was reanalyzed with improved colocalization of the pediatric and adult controls in the Main Cohort (Sup Fig 6G-H) and in the Reproducibility Cohort (Sup Fig 7B&E). Filtering storage-sensitive molecules out of lipidomics and LM datasets was impossible due to the ubiquity of oxidation-sensitive lipids and LMs. To maintain the ability to compare patients to healthy controls while guarding against patterns that may be driven by non-disease differences between pediatric and adult samples, pediatric samples were included for PCAs to identity vectors that exclusively described disease patterns of variance but were excluded from direct parallel univariate statistical analysis. In addition, correlates of disease identified by parallel univariate analysis were further validated against pediatric controls throughout the analysis described here to estimate robustness.

**Assessment of the Resolution of Previous pRD subtypes in Omics Datasets:**

Until recently pRD was further differentiated into Leaky SCID (LS), and delayed onset combined immune deficiency (CID). Recently the differentiation of these two groups has been abandoned due to symptom overlap and the lack of clinical utility in informing treatment. Among the goals of this study was the establishment of new diagnostic criteria with which to characterize. To assess the ability of these data to differentiate the previously used pRD-CID and pRD-LS subtypes, we assigned these previous designations to the pRD patients in our Main Cohort. The previously published symptom distribution between pRD-LS and pRD-CID failed to differentiate these subtypes (Sup Fig 8).(3) No separation was observable along the PCs that separated OS and SCID or on PCs that showed pRD-driven separation from controls (Sup Fig 8). To investigate if any metabolite, lipid, or LM was significantly different between pRD-CID and pRD-LS we compared each dataset with a Mann-Whitney U with correction for multiple comparisons set at a false discovery rate of 10 % using the Benjamini-Hochberg method. No metabolite, lipid, or LM passed this criterion, supporting the conclusion that pRD-CID and pRD-LS are not distinguishable in omics datasets analyzed here.

**Supplemental Figures and Captions:**

**Supplemental Figure 1: Metabolomics yields OS and SCID dominant patterns.** Cross RAG-disease subtypes and adult controls using a Kruskal-Wallis test. All metabolites displayed pass a correction for multiple comparisons at a 10 % FDR via the Benjamini-Hochberg method. The size of each node displays the negative logarithm of the p-value for each subtype compared to the adult healthy control samples via a Mann-Whitney U test. The color of each node displays the base two logarithm of the fold change of the indicated subtype relative to the adult healthy controls. This plot corresponds to Figure 2A and displays all metabolites that pass FDR 10% inclusion criteria.

B

A

D

C

**Supplemental Figure 2: Metabolomics from reproducibility cohort supports Trp and XOR dysregulation in RAG deficiency. (A)** Cross group metabolite comparison of RAG-disease subtypes versus adult controls using a Kruskal-Wallis test. Metabolites displayed pass a correction for multiple comparisons at a 10 % FDR via the Benjamini-Hochberg method. **(B)** Trp-metabolism map for each RAG-disease subtype compared to the adult healthy controls. **(C)** XOR metabolic map for each RAG-disease subtype compared to the adult healthy controls. **(D)** Scatter plots of representative Trp and XOR metabolites including pediatric control samples with the median of each group displayed. Data in **(D)** have been log-transformed and z-scaled for visualization.

**Supplemental Figure 3: PUFA-derived LMs separate disease subtypes. (A)** Cross group LM comparison including RAG-disease subtypes and adult controls from the main cohort using a Kruskal-Wallis test. LMs displayed pass a correction for multiple comparisons at a 10 % FDR via the Benjamini-Hochberg method. LMs are organized by the enzymatic process that produces them. Plot shows the average base two logarithm of the fold change as node color and the negative logarithm of the Mann-Whitney U raw p-value as node size for each group vs. the adult controls.

A

C

B

**Supplemental Figure 4: Oxidized lipid mediators from reproducibility cohort show increased free PUFAs and hydroxylated lipid products. (A)** Cross group LM comparison including RAG-disease subtypes and adult controls from the reproducibility cohort using a Kruskal-Wallis test. LMs displayed pass a correction for multiple comparisons at a 10 % FDR via the Benjamini-Hochberg method. LMs are organized by the enzymatic process that produces them. **(B)** Pathway maps of Arachidonic acid (AA) and Docosahexaenoic acid (DHA) with LM metabolites shown as circles and enzymes shown as hexagons. Each node displays the average base two logarithm of the fold change as node color and the negative logarithm of the Mann-Whitney U raw p-value as node size for each group vs. the adult controls.  **(C)** Scatter plot of representative LMs from 12-LOX and 15-LOX pathways including in pediatric control samples with the median of each group displayed. Data in (C) have been log-transformed and z-scaled for visualization.

**Supplemental Figure 5: Metabolite and LM correlates of clinical measures in the reproducibility cohort.** Pairwise nonparametric correlation network analysis of the lipidomic dataset from the reproducibility cohort mapping only first and second order neighbors of the RAG subtype critical element list (CD3, CD4, CD8, anti-IFN-α, anti-IFN-β, anti-IFN-ω, IL-6, IL-7, IL-8, IFN-γ, and CCL17). (**A**) Complete network map of with all displayed connections reflecting an absolute value greater than 0.6 for the correlation coefficient and p-value less than 0.01. (**B**) Zoomed region of the same network shown in (A) showing absolute immunoglobulin and autoreactive immunoglobulin interactions with metabolites including bile acids and Trp-metabolites. (**C**) Zoomed region of the same network shown in (A) showing lymphocyte number correlates in the metabolomics data.

B

E

H

F

C

G

D

A

**Supplemental Figure 6: Pediatric control sample decay limits direct statistical comparison in the Main Cohort. (A-C)** Principal Component Analysis (PCA) of each of the datasets measured here and the corresponding Loading Plots (**D-F**). Metabolomics and LM data was z-scaled while lipidomics data was pareto scaled to avoid overweighting of low abundance lipids within each class. Metabolites, lipids, and LMs are colored by their molecular class or pathway. Abbreviations: Amino acids (AA), Tricarboxylic Acid Cycle (TCA), Lysophospholipids (Lysos), Phosphatidylcholine (PC), Phosphatidylethanolamine (PE), Phosphatidylglycerol (PG), Phosphatidylinositol (PI), Phosphatidylserine (PS), Sphingolipids (Sphingos), 12-Lipoxygenase (12-LOX), 15-Lipoxygenase (15-LOX), 5-Lipoxygenase (5-LOX), Cyclooxygenase (COX), Cytochrome P450 (CYP450), Free Polyunsaturated Fatty Acid (PUFA). (**G-H)** PCA and corresponding loadings plots demonstrate that the removal of energized molecules from the metabolomics dataset partially circumvents sample decay limitations.

D

E

F

C

B

A

**Supplemental Figure 7: Reproducibility Cohort Pediatric control sample aging limits direct statistical comparison in the reproducibility cohort.**  **(B**-**G**) Principal Component Analysis (PCA) of each of the datasets measured here including the scores plots and the corresponding loading plots (E-G). Abbreviations: Amino acids (AA), Tricarboxylic Acid Cycle (TCA), 12-Lipoxygenase (12-LOX), 15-Lipoxygenase (15-LOX), 5-Lipoxygenase (5-LOX), Cyclooxygenase (COX), Cytochrome P450 (CYP450), Free Polyunsaturated Fatty Acid (PUFA).

A

B

C

D

E

F

**Supplemental Fig 8: Metabolite, Lipid, and LM datasets cannot distinguish previous CID and LS pRD subtypes.** (**A-C**) Principal Component Analysis (PCA) plots of each of the datasets measured here and (**D-E**) the corresponding loading plots. Abbreviations: Amino acids (AA), Tricarboxylic Acid Cycle (TCA), 12-Lipoxygenase (12-LOX), 15-Lipoxygenase (15-LOX), 5-Lipoxygenase (5-LOX), Cyclooxygenase (COX), Cytochrome P450 (CYP450), Free Polyunsaturated Fatty Acid (PUFA).

**References:**

1.	Garwolińska D, Młynarczyk M, Kot-Wasik A, Hewelt-Belka W. The influence of storage on human milk lipidome stability for lipidomic studies. Journal of proteome research. 2021;21(2):438–46.

2.	Reis GB, Rees JC, Ivanova AA, Kuklenyik Z, Drew NM, Pirkle JL, et al. Stability of lipids in plasma and serum: Effects of temperature-related storage conditions on the human lipidome. Journal of Mass Spectrometry and Advances in the Clinical lab. 2021;22:34–42.

3.	Bosticardo M, Dobbs K, Delmonte OM, Martins AJ, Pala F, Kawai T, et al. Multiomics dissection of human RAG deficiency reveals distinctive patterns of immune dysregulation but a common inflammatory signature. Science immunology. 2025;10(103):eadq1697.

4.	Castagnoli R, Pala F, Subramanian P, Oguz C, Schwarz B, Lim AI, et al. Immunopathological and microbial signatures of inflammatory bowel disease in partial RAG deficiency. Journal of Experimental Medicine. 2025;222(8):e20241993.

5.	Lemons J, Conrad M, Tanes C, Chen J, Friedman E, Roggiani M. Enterobacteriaceae growth promotion by intestinal acylcarnitines, a biomarker of dysbiosis in inflammatory bowel disease. Cell Mol Gastroenterol Hepatol. 2024; 17 (1): 131–48.
